# Successful awake total thyroidectomy using a bilateral intermediate cervical plexus block technique in a high-risk patient: a challenge for the anesthesiologists’ case report

**DOI:** 10.1097/MS9.0000000000003161

**Published:** 2025-03-19

**Authors:** Amal A. Abu Jheasha, Mohammad Alballasi, Tasneem Alsharif, Ahmad Ruzyqat, Mohammad Atawneh, Sami Bannoura, Falah Ibedo

**Affiliations:** a Alia governmental Hospital, Hebron, State of Palestine; b Al-Quds University-School of Medicine, Abu-Dis, East Jerusalem; c Department of Anesthesia, Department of Pathology, Al- Ahli Hospital, Hebron, State of Palestine

**Keywords:** American Society of Anesthesiologists, ankylosing spondylitis, awake thyroidectomy, bilateral cervical plexus block, case report, local anesthesia, Mallampati score

## Abstract

**Introduction:**

Ankylosing spondylitis (AS), a chronic inflammatory arthritis, affects the spine and sacroiliac joints. Awake thyroidectomy is a safe operation for high-risk patients, with local anesthesia often needed.

**Importance:**

The choice between general and loco-regional anesthetic techniques is challenging due to technical difficulties in high-risk patients.

**Case presentation:**

A 52-year-old male patient with neck pain and anterior neck swelling was diagnosed with thyroid cancer. Ultrasound scans revealed bilateral thyroid nodules, and a total thyroidectomy was performed under regional anesthesia. A patient with severe ankylosing spondylitis (AS), lung fibrosis, and Grade 3 Mallampati score underwent total thyroidectomy under local anesthesia using the bilateral intermediate cervical plexus block technique (BICPB).

**Clinical discussion:**

The patient’s condition, including AS and other comorbidities, made intubation difficult due to vertebral bone fusion, back and neck flexibility loss, and possible temporomandibular joint disease. The patient’s high American Society of Anesthesiologists (ASA) Classification System led to the use of local anesthesia with BICPB, avoiding general anesthesia with placed endotracheal tube (ET tube). The intermediate cervical plexus block (ICPB) technique is a non-invasive procedure that reduces postoperative respiratory events and improves outcomes. It is particularly effective in patients with lung fibrosis. However, concerns about potential complications, such as bilateral phrenic nerve palsy, respiratory failure, and infections, persist.

**Conclusion:**

This case report highlights the use of bilateral cervical plexus block (BICPB) in patients with ankylosing spondylitis and other comorbidities after a total thyroidectomy. This method is safer and more effective than general anesthesia, providing pain-free periods and reducing the need for analgesics and opioids. However, anesthesiologists must consider the disease extension, upper airway involvement, positioning difficulties, and technical difficulties when planning anesthesia management.

## Introduction

Ankylosing spondylitis (AS), also known as Bechterew disease or Marie Strumpell disease, is a painful chronic inflammatory arthritis characterized by exacerbations (flares) and periods of quiescence. It mostly affects the spine and sacroiliac joints^[1]^.

Patients with chronic spine illnesses provide unique problems for anesthesiologists, and special precautions should be taken to avoid excessive neck manipulation, which may induce spinal cord damage. Despite the existence of restricted airways, most anesthesiologists opt to employ general anesthesia in those patients rather than the neuroaxis^[2]^.

**HIGHLIGHTS**

- Ankylosing spondylitis (AS) is a chronic inflammatory arthritis that required the patient to undergo a total thyroidectomy under regional anesthesia.
- The patient with severe AS, lung fibrosis, and a Grade 3 Mallampati score encountered challenges during intubation; however, the bilateral intermediate cervical plexus block technique effectively addressed these difficulties.
- The BICPB technique minimizes complications associated with general anesthesia, particularly benefiting patients with lung fibrosis. Nonetheless, it is crucial for anesthesiologists to remain alert for possible complications such as phrenic nerve palsy and infections, demonstrating the technique’s value in total thyroidectomy procedures.

Thyroidectomy is often performed under general anesthesia with endotracheal intubation, although in high-risk patients, regional anesthesia with monitored anesthesia care is safer than general anesthesia and may be provided to select thyroidectomy candidates^[3]^.

Regional or neuraxial anesthesia should be considered an optimal technique for the avoidance of general anesthesia-related complications when indicated in pulmonary fibrosis patients^[4]^.

Awake thyroidectomy is a well-tolerated and safe operation for adequately chosen individuals, especially those patients who are classified as high-risk cases which cannot tolerate general anesthesia due to their co-morbidities, with several potential advantages over general anesthesia. In most circumstances, just local anesthesia is needed.

Better familiarity with this method may lead to better patient comfort^[5]^.

## Case presentation

A 52-year-old male patient presented complaining of neck pain and anterior neck swelling for six years. These symptoms were of gradual onset and progressive in course, the swelling had grown larger in the last 5 months and became associated with dysphagia and hoarseness of voice which make the patient to seek medical advice for, and the patient denied a history of unintended weight loss.

His past medical history included longstanding ankylosing spondylitis, ischemic heart disease requiring catheterization threee times for non-obstructive coronary artery disease, type 2 diabetes, and lung fibrosis requiring intermittent home oxygen therapy, gout, and hyperlipidemia and known to have allergy to metronidazole and non-steroidal anti-inflammatory drugs (NSAIDs).

Physical evaluation revealed an afebrile patient, not in respiratory distress with mild hoarseness of voice. His heart rate was regular at 78 bpm, blood pressure was 123/85 mmHg, and SpO_2_ was 92% on room air. On neck examination, there was an oval-shaped central neck swelling, tender to palpation, and moving with swallowing. No hotness or redness or skin changes appear. The patient was found to have a high-grade Mallampati score. Chest auscultation yielded a diffused expiratory wheeze and fine crepitation in lower lung fields; examination of other systems was unremarkable except for limited neck movement due to his chronic posterior neck pain.

Upon admission, appropriate laboratory work-up was done. Hematological and serum biochemistry was essentially within the normal range including the thyroid profile which revealed the euthyroid state (Table 1).

**Table 1:** Laboratory work-up

Variables	Reference range for this hospital	Result upon Admission
White-cell count (per mm^3^)	5000̄̄–10 000	6500
Neutrophils (per mm^3^)	2500–7500	2150
Lymphocytes (per mm^3^)	1500–3500	2780
Monocytes (per mm^3^)	40–800	840
Basophils (per mm^3^)	15–100	160
Eosinophils (per mm^3^)	40–440	580
Hemoglobin (g/dl)	12–16	15.9
Hematocrit (%)	37–48	40
Platelet count (per mm^3^)	150 000–400 000	186 000
Prothrombin time (sec)	11–14	13.1
International normalized ratio	0.9–1.1	1.01
Activated partial-thromboplastin time (sec)	25–36	23
Sodium (mmol/liter)	132–148	140
Potassium (mmol/liter)	3.9–5.7	3.9
Chloride (mmol/liter)	96–109	109
Creatinine (mg/dl)	0.4–1.1	1.17
Urea nitrogen (mg/dl)	4.7–23.4	19
Aspartate aminotransferase (U/L)	0–45	27
Alanine aminotransferase (U/L)	0–31	13
Alkaline phosphatase (U/L)	Up to 105	77
Total bilirubin (mg/dl)	Up to 1.2	0.4
HbA1C (%)	<5.7	5.2
TSH (IU/ml)	0.4–4.94	1.4
FT3	1.45–3.5	3.1
FT4	0.7–1.5	1.4

Imaging required was an ultrasound scan of the neck which showed bilateral thyroid nodules involving mainly the left lobe. The right thyroid lobe was enlarged with three nodules: the largest one measuring 2.7*1.3 cm, well-defined, isoechoic, with solid components and few foci of calcification surrounded by a halo rim sign and appearing wider than taller. Likewise, the left lobe was enlarged with well-defined, heterogeneous solid, cystic, and isoechoic nodules containing foci of calcification inside which measured 4*2.8 cm with TIRDA 4. An ultrasound scan showed that the left lobe nodule started to invade the surrounding muscles.

Ultrasound-guided FNA was performed showing features in keeping with papillary, neoplasm, Bethesda system category IV. The diagnosis was made. The patient was offered total thyroidectomy by the surgery team which he gave consent for.

Based on the patient’s history and age, ECHO and ECG were performed and were within the normal range.

During preanesthetic evaluation, the patient was informed about the anesthetic and surgical steps, especially the regional anesthesia technique, and he was awake during the entire surgery. He was instructed to try to remain calm and immobile to avoid unnecessary coughing and swallowing. No premedication was administered preoperatively.

Intraoperative monitoring of the electrocardiogram, noninvasive blood pressure, pulse oximeter, and end-tidal carbon dioxide was conducted. In the operating room, the patient received cefazolin 2 gram as antibiotic prophylaxis. Oxygen was delivered via a simple face mask at 6 L/min.

Total thyroidectomy was done under regional anesthesia (ultrasound-guided bilateral intermediate cervical plexus block); the patient was classified to be difficult to intubate because the patient had ankylosing spondylitis associated with marked neck stiffness. This, in turn, impedes the process of endotracheal intubation (ETT) which is essential to keep the airway open during the surgery.

**Figure 1.**
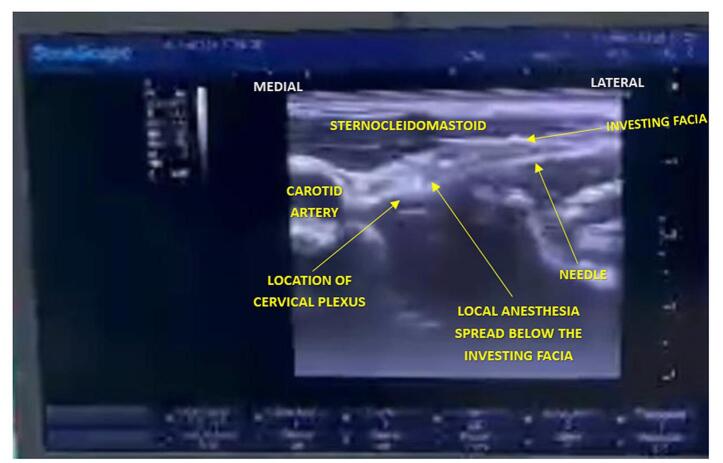
The investing fascia in the intermediate approach to the cervical plexus is plainly identifiable, and LA is deposited beneath the fascia.

The patient is typically positioned supine or semi-recumbent, with his head turned away from the injection site. After cleaning the skin, the ultrasound transducer is placed on the lateral side of the neck, aligning with the midpoint of the sternocleidomastoid muscle. The needle is inserted perpendicular to the skin until resistance is felt, indicating passage through the cervical fascia. Local anesthetic is then slowly injected while monitoring its spread along the posterior border of the sternocleidomastoid muscle using ultrasound imaging (Fig. 1). This technique ensures precise placement and effective anesthesia; total thyroidectomy was then done without any immediate or post-operative complications.

The patient was able to be discharged from the hospital the following day, with the patient experiencing pain-free recovery without the need for analgesic medications and without any notable complications.

**Figure 2.**
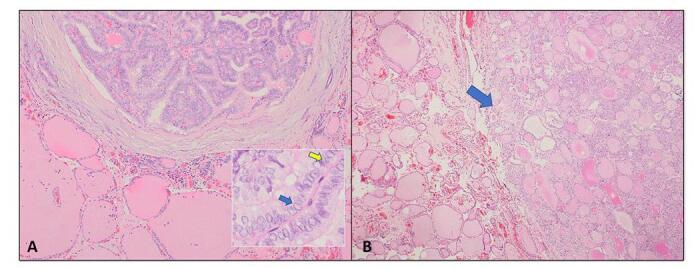
Papillary thyroid microcarcinoma and multinodular goiter. **A.** Section shows an incidental focus of papillary thyroid microcarcinoma (H&E, 10X); the insert shows cellular crowding with typical nuclear features of papillary carcinoma including nuclear enlargement, grooves (yellow arrow), and chromatin clearing (blue arrow). **B.** Hyperplastic thyroid nodule (blue arrow) (H&E, 4X).

Pathology of the specimen of the thyroid gland revealed papillary thyroid micro carcinoma, 1 mm limited to the right thyroid lobe, multinodular goiter with frequent hyperplastic nodules, and negative surgical margins (Fig. 2).

## Discussion

AS is a chronic inflammatory disease that affects the axial and peripheral joints. Its main feature is vertebral bone fusion, which causes back and neck flexibility loss. Thus, AS patients pose the greatest challenge^[6–8]^. These patients have the most serious intubation and difficult airway issues imaginable, due to decreased or no cervical spine mobility, fixed flexion deformity of the thoracolumbar spine, and possible temporomandibular joint disease. Sound clinical judgment is critical for timing and selecting the method of airway intervention and anesthesia technique. The choice between general and loco-regional anesthetic techniques is not easy because both present technical difficulties in achieving them, that is why a discussion is required between the patient, surgeon, and anesthesiologist about the potential risks and benefits of regional and general anesthesia^[9,10]^. Regional anesthesia (cervical plexus block) offers many advantages over general anesthesia in these patients, but cervical plexus blocks are known to be difficult, though not impossible, depending on the severity of the disease like our patient. In our case, the trend has been to deal with local anesthesia with BICPB and avoid general anesthesia with placed endotracheal tube (ET tube) as in our patient who is considered by the high American Society of Anesthesiologists (ASA) Classification System.

Before starting, light sedation with dexmedetomidine is administered to minimize patient stress while maintaining responsiveness and cooperation throughout the procedure.

**Figure 3.**
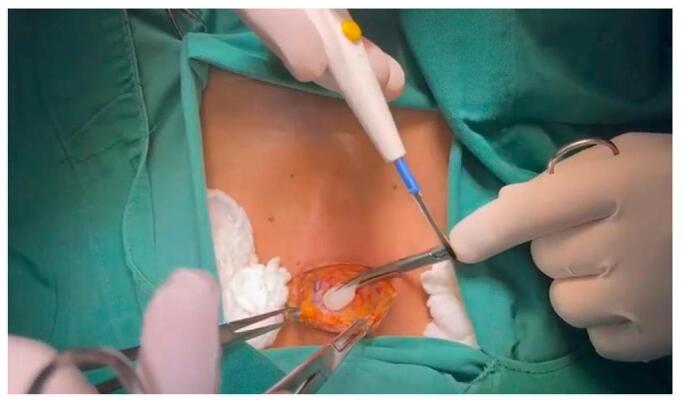
A surgical incision is made through the skin and subcutaneous fat, extending 2 to 3 cm just superior to the suprasternal notch, between the two sternal heads of the sternocleidomastoid muscle.

**Figure 4.**
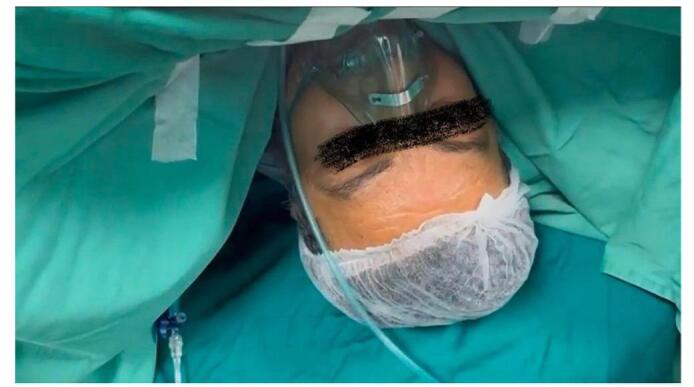
The patient is awake and breathing spontaneously through an oxygen face mask while the surgery is ongoing.

Intermediate cervical plexus block (ICPB), an ICPB technique, involves a slightly more posterior superficial cervical block at C4. LA is inserted between the superficial and deep cervical fascia in the neck’s posterior triangle. In our patient, the block is performed under ultrasound guidance as follows: to begin with, we placed a small linear ultrasound probe in the posterior triangle of the neck near the transverse process of C4. The needle was then inserted anterior-to-posterior through the SCM and past the prevertebral fascia in a plane with the transducer. The posterior cervical space is bordered by prevertebral fascia superficially, deep cervical paravertebral fascia medially, middle scalene or Levator scapulae muscles posteriorly, and longus capitis or anterior scalene muscles anteriorly (Fig. 3 & 4).

- After negative aspiration, we injected 15 mL of LA in 5 mL increments, gently aspirating between injections. LA imagined spreading throughout the space described above (an ultrasound-guided intermediate CPB was performed using the posterior approach. Using a 16 MHz linear probe and a 22 G × 2” non-insulated needle (ultaraplex 360; B. Braun, Germany), a volume of 15 mL local anesthetic (10 mL of bupivacaine 0.5%, 4 mL of lidocaine 1%, and 1 mL dexamethasone 4%) was injected in the posterior cervical space (between the sternocleidomastoid muscle and the prevertebral fascia) as described by Choquet et al.4 (Fig. 1). The same procedure is done on the opposite side of the neck to complete a bilateral intermediate CPB).

After CPB, total thyroidectomy was done when the patient was awake; the procedure was completed without any complications, as the patient was responsive and interactive throughout the procedure and after its completion. The patient remained under observation and was monitored for two days, where he remained stable without any complaints or complications; then, he was discharged from the hospital in a stable health condition. Cervical plexus block is more effective than GA at reducing postoperative respiratory events and improving outcomes by blunting the stress response. In difficult airways, cervical plexus block anesthesia provides several advantages, including diminished airway handling. Hemodynamic stability is another frequently mentioned characteristic^[11–16]^. Furthermore, it reduces the likelihood of pulmonary complications in patients with lung fibrosis.

Furthermore, cervical plexus block anesthesia has excellent postoperative analgesic properties and reduces nausea and vomiting. As a result, patients recover faster and spend less time in the hospital. Overall, the uncommon use of intermediate cervical plexus blocks for neck surgeries may be due to some concern about potential complications such as bilateral phrenic nerve palsy and respiratory failure, inadvertent intravascular injection of local anesthesia, spinal cord injury, infections, cardiovascular depression, and failed block. Regardless, these complications could be avoided with experience and precautions. Moreover, a preoperative respiratory function evaluation and the patient’s condition optimization are required.

## Conclusion

Bilateral cervical plexus block (BICPB) is not commonly used in surgical settings due to certain limitations and potential complications. However, this particular case illustrates its effectiveness for a patient with ankylosing spondylitis (AS) and other comorbidities who underwent total thyroidectomy. BICPB can serve as a safer and more efficient alternative to general anesthesia, leading to reduced postoperative pain and less reliance on opioids. This situation showcases how BICPB could broaden anesthetic options for both complex and traditional surgeries. Nevertheless, careful anesthetic planning is crucial, taking into account the patient’s disease severity, possible involvement of the upper airway, challenges with positioning, and any technical difficulties. For patients with AS and significant cardiopulmonary issues, anesthetic risks are heightened, which calls for tailored management strategies and the expertise of skilled professionals. While BICPB has its advantages, the technical challenges associated with it might elevate the risk of complications, emphasizing the need for thorough evaluation on a case-by-case basis.

## Methods

The work has been reported in line with the SCARE 2023 criteria^[17]^.

## Data Availability

Not available.
